# Laser‐Induced 3D Graphene Enabled Polymer Composites with Improved Mechanical and Electrical Properties Toward Multifunctional Performance

**DOI:** 10.1002/advs.202509039

**Published:** 2025-09-03

**Authors:** Fu Liu, Sida Luo, Jingyang Li, Zhe Wang, Xu Wang, Wenqian Hao, Yanan Wang

**Affiliations:** ^1^ School of Aerospace Engineering North University of China Taiyuan 030051 China; ^2^ Shanxi Engineering Research Center of Unmanned Aerial Vehicle North University of China Taiyuan 030051 China; ^3^ School of Mechanical Engineering & Automation Beihang University No. 37 Xueyuan Road Beijing 100191 China; ^4^ Beijing Tsing Aero Armament Technology Co., Ltd. Beijing 100190 China; ^5^ AVIC Research Institute for Special Structures of Aeronautical Composite Ji'nan 250023 China

**Keywords:** flexible sensors, laser‐induced 3D graphene, multifunctional structures, polymeric composites

## Abstract

Conductive graphene‐based composites are attracting substantial interest due to their excellent mechanical and electrical properties for potential applications in electronics. Typically, such composites are fabricated by infiltrating the 3D graphene framework with the polymer matrix. However, the production of 3D graphene foams is limited by the challenges in preparing graphene dispersions, while 3D printing presents a significant breakthrough in the fabrication of desired 3D graphene‐based structures. Here, the utilization of the 3D printing technique driven by laser‐induced graphene (LIG) and a conventional penetration process for assembling graphene‐based conductive polymer composites is described. The synergistic integration endowed the proposed 3D‐LIG/epoxy composites with an electrical conductivity of 3.54 S m^−1^ in through‐plane, and a tensile strength of ≈5.4 MPa by a 7606% improvement with a high specific strength of 6.8 × 10^3^ (N m) kg^−1^. Meanwhile, the flexible composites exhibited an outstanding ductility reaching a 230% tensile‐failure strain and a 50% high linear elastic strain. The prepared 3D‐LIG/polymer composites thus demonstrated the functionalized behaviors for applications in de‐icing, microwave absorption, and flexible sensors.

## Graphical abstract

In this study, the utilization of 3D printing driven by laser‐induced graphene and a conventional infiltration process for assembling kinds of graphene‐based polymer composites is described. The synergistic integration of polymers and the interconnected 3D‐LIG frameworks has endowed the composites with remarkable mechanical, electrical, and tunable properties for applications in de‐icing, microwave absorption, and flexible sensors.

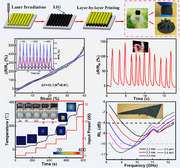

## 1 Introduction

Macroscopic 3D graphene (3DG) architectures assembled by the 2D graphene materials introduce enhanced features, such as the huge specific surface area,^[^ ^1^ ^]^ remarkable electrical conductivity^[^ ^2^ ^]^ and thermal properties,^[^ ^3^ ^]^ which enable the efficient and sufficient usage of 2D graphene sheets in practical applications.^[^ ^4^ ^]^ 3D graphene‐based composites, one of the 3DG architectures, integrate the properties of graphene and polymers, presenting excellent properties in mechanics and electricity, as well as great emerging applications in flexible electronics, strain sensors, and electromagnetic functional materials.^[^ ^5^, ^6^, ^7^ ^]^ According to basic synthesizing principles of conductive 3DG composites, two overarching types have been loosely categorized into: the mixing method prepared by dispersing/mixing conductive nanomaterials into polymers, and the dip‐coating method with formed 3D conductive structures directly applied to immerse in the polymer matrix.^[^ ^8^, ^9^ ^]^ For example, Chen et al.^[^ ^10^ ^]^ and Zhang et al.^[^ ^11^ ^]^ reported the development of graphene‐based composites where the graphene nanostructures were mixed and incorporated with the polymer matrix as the forming elements for the 3D architectures. Song et al.^[^ ^12^ ^]^ and Xiao et al.^[^ ^13^ ^]^ utilized macroporous graphene foams synthesized by chemical vapor deposition (CVD) as the 3D conductive skeletons to manufacture graphene/PDMS and graphene/resin composites through infiltration, respectively. Similarly, by usage of the graphene‐based ink/gels/resins, macrostructure components were fabricated layer‐by‐layer through 3D printing, such as ink printing,^[^ ^14^ ^]^ extrusion printing,^[^ ^15^ ^]^ stereolithography,^[^ ^16^ ^]^ etc. In comparison, the porous networks of the 3DG foams need not be dispersed in polymers when preparing composites, and remain completely separated from the polymer matrices, preserving the inherent properties of the resin. However, the complexity of the template‐assisted method or self‐assembly method seriously limits the production and realistic development of the 3DG based architectures.^[^ ^17^, ^18^ ^]^ Thus, the ability to effectively prepare a 3D graphene structure with controllable designs is desirable for both academic research and industrial applications of the 3DG composites.

As a one‐step, high‐efficiency, and flexible process, the laser‐induced graphene (LIG) technique has been developed for synthesizing graphene with controllability in fabrication, structure, and property.^[^ ^19^ ^]^ With programmed processing steps, the LIG technique has evolved into an indispensable processing platform upon which graphene‐related innovative materials and devices have been successfully exploited by combining with traditional techniques or materials,^[^ ^20^, ^21^, ^22^ ^]^ and the obtained LIG structures have been applied to electronics,^[^ ^23^ ^]^ water desalination,^[^ ^24^ ^]^ energy storage^[^ ^25^ ^]^ and biological sensors.^[^ ^26^ ^]^ By leveraging this versatility, various intelligent LIG‐based polymer composites have been produced, including self‐sensing composites for strain monitoring,^[^ ^27^, ^28^, ^29^ ^]^ multifunctional surfaces/interfaces for microwave shielding and absorption,^[^ ^30^, ^31^, ^32^ ^]^ and self‐protection.^[^ ^33^, ^34^ ^]^ Although the functional composites are achievable, the obtained LIG structures are mainly integrated on the surface or interlamination of the above‐mentioned composites, resulting in the anisotropy of structure and property due to the dependency of the film/paper‐derived LIG to the substrate. To alleviate this situation and further promote the development of LIG composites, the free‐standing 3D LIG architectures were synthesized for the fabrication of the LIG‐based functional composites.^[^ ^35^, ^36^ ^]^ Specifically, Luong^[^ ^37^ ^]^ and Song et al.^[^ ^38^ ^]^ utilized the additive manufacturing methods for the direct fabrication of 3D LIG foams with porous structures based on laser‐induced graphene and laminated objected manufacturing (LOM). For assembly of 3D composites, Luo et al.^[^ ^39^ ^]^ combined the LIG technique with a hot‐pressing process to fabricate smart polymer honeycombs for electromagnetic shielding and stealth. With the same strategy, Kang et al.^[^ ^40^ ^]^ prepared functionalized LIG interfaces for improved anisotropy and photo‐electro‐thermal performance on the polymeric composites by the FDM technique. However, these methods are still constrained by the requirement for the templates and adhesives, which limits the controllability and design flexibility in the fabrication of 3D graphene‐based architectures. Thus, a facile LIG process enabling straightforward freeform manufacturing of 3D graphene‐based composites has yet to be reported.

Following this line of thought, a LIG‐assisted additive manufacturing protocol we developed has been employed to directly print 3D‐LIG foams with desired patterns and structures, eliminating the need for additional binders, templates, or catalysts.^[^ ^41^ ^]^ Using the as‐prepared graphene foam as a porous scaffold, various graphene‐based composites are fabricated by a simple penetration process. On the basis of the forming mechanism, the free‐standing 3D‐LIG foams with porous networks and a huge specific surface area have been produced by dissolving the sintered polymer scaffolds within the printed specimens. The interconnected and open pathways within the scaffold allow for the infiltration of the polymer matrix to assemble the composites. The integration of 3D‐LIG foams with polymer matrix has endowed the resulting composites with exceptional electrical properties. Meanwhile, the synergy has exhibited a boost in the mechanical properties of 3D‐LIG foams. Along with the flexible fabrication, controllable structures, and integrated performance, the as‐prepared 3D‐LIG composites demonstrate multiple advanced functionalities, showing great potential for applications in flexible sensors, heaters, and microwave absorption devices.

## 2 Results and Discussion

### 2.1 Fabrication of the 3D‐LIG Architectures

**Figure 1:**
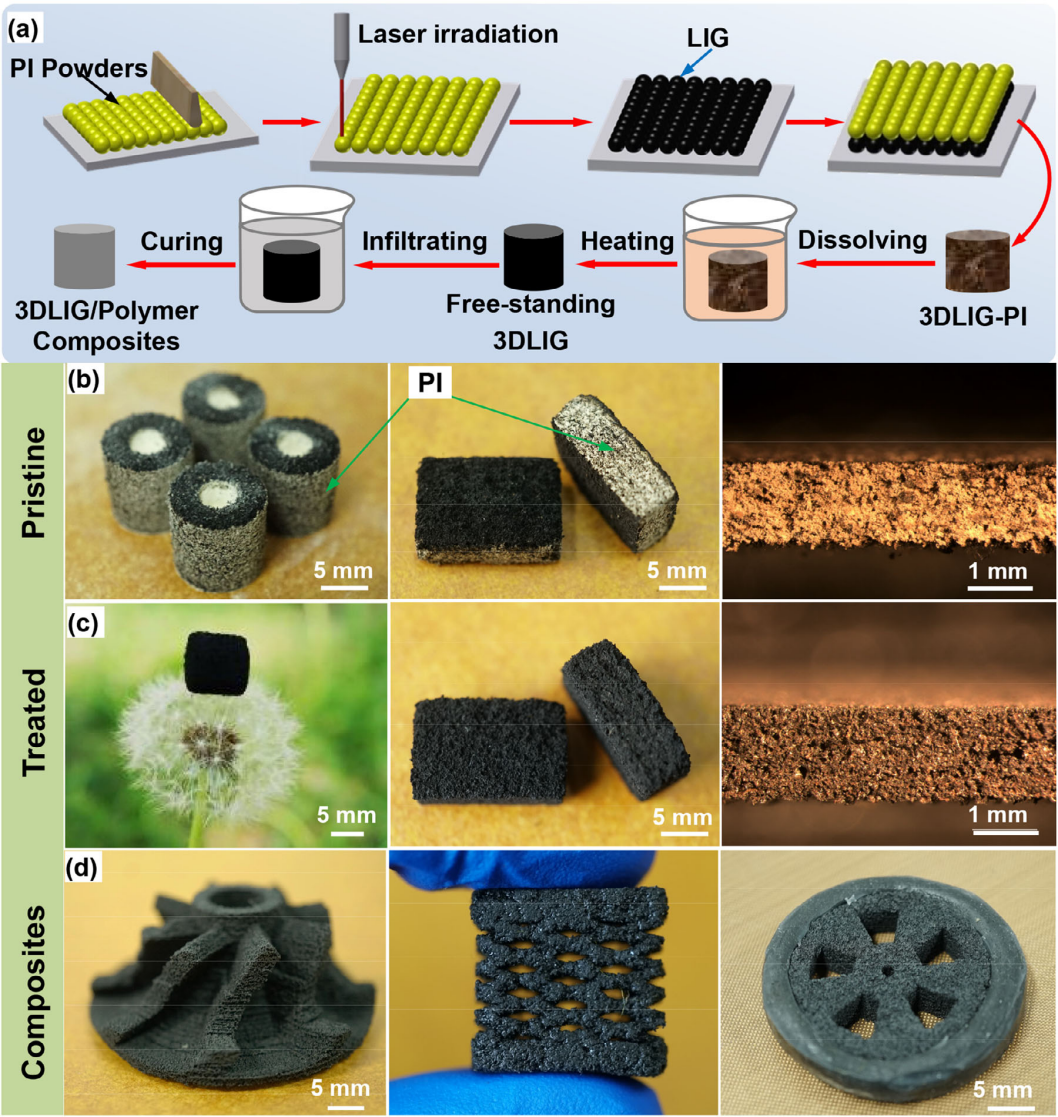
Manufacturing of 3D graphene‐based architectures using laser‐induced graphene. (a) Schematic description of the fabrication of the 3D‐LIG architectures; The photographs and the macroscopic cross‐section morphology of the pristine 3D‐LIG foams (b) and free‐standing 3D‐LIG foams (c); (d) The obtained rubber and epoxy‐filled 3D‐LIG composites.

The processing procedure of the 3D‐LIG architectures is schematically depicted in **Figure** **1**. Figure 1a describes the whole preparation scheme for the free‐standing 3D‐LIG foams and 3D‐LIG‐based composites. By programmed controlling the cross‐sectional patterns of each layer, the designed 3D‐LIG foams have been printed layer‐by‐layer using the selective laser carbonizing (SLC) technique on PI powders, which serve both as the carbon source and the template. In order to introduce more porous structures, the obtained foams are soaked in N, N‐Dimethylformamide (DMF) to dissolve the sintered PI scaffolds. Subsequent to the dissolution, the 3D‐LIG composites are manufactured by immersing the free‐standing LIG foams into the polymer solution following the procedure described in Experimental Part. Photographs and optical micrographs in Figure 1b,c compare the macro‐morphology of the specimens before and after treatment. As exhibited, the sides of the cylinder and cube specimens are covered by yellowish sintered PI particles, while the specimens subjected to the dissolving completely reveal black carbon structures in the whole surfaces. Simultaneously, the optimized specimens maintain the macroscopic contour of the pristine ones without deformation and shrinkage, and perform a 20 g cm^−3^ ultra‐low density and a porosity of 99%, which can freely stand on the dandelion without any bending in Figure 1c. The obtained 3D‐LIG/epoxy composites and 3D‐LIG/elastomer composites, and their assembly component of a wheel model, are demonstrated in Figure 1d. The contour of the turbine model assembled by epoxy composites keeps consistent with the geometry of the pristine 3D‐LIG foams, revealing the perfect combination of graphene and polymer. As a smart electronic material, the elastomer composites with the kirigami feature can serve as piezoresistive sensors and artificial skins. The wheel model composed of both epoxy and rubber composites intuitively illustrates the flexibility of the SLC process for fabricating arbitrary 3D graphene structures. Briefly, the SLC printing technique is convenient and efficient with no high‐temperature furnace employed and no multi‐step self‐assembly applied, enabling the direct production of various designed structures with desired features for 3D‐LIG architectures, providing sufficient source materials for synthesizing 3D‐LIG derivatized composites.

### 2.2 Morphological and Structural Characterizations of the 3D‐LIG Architectures

**Figure 2:**
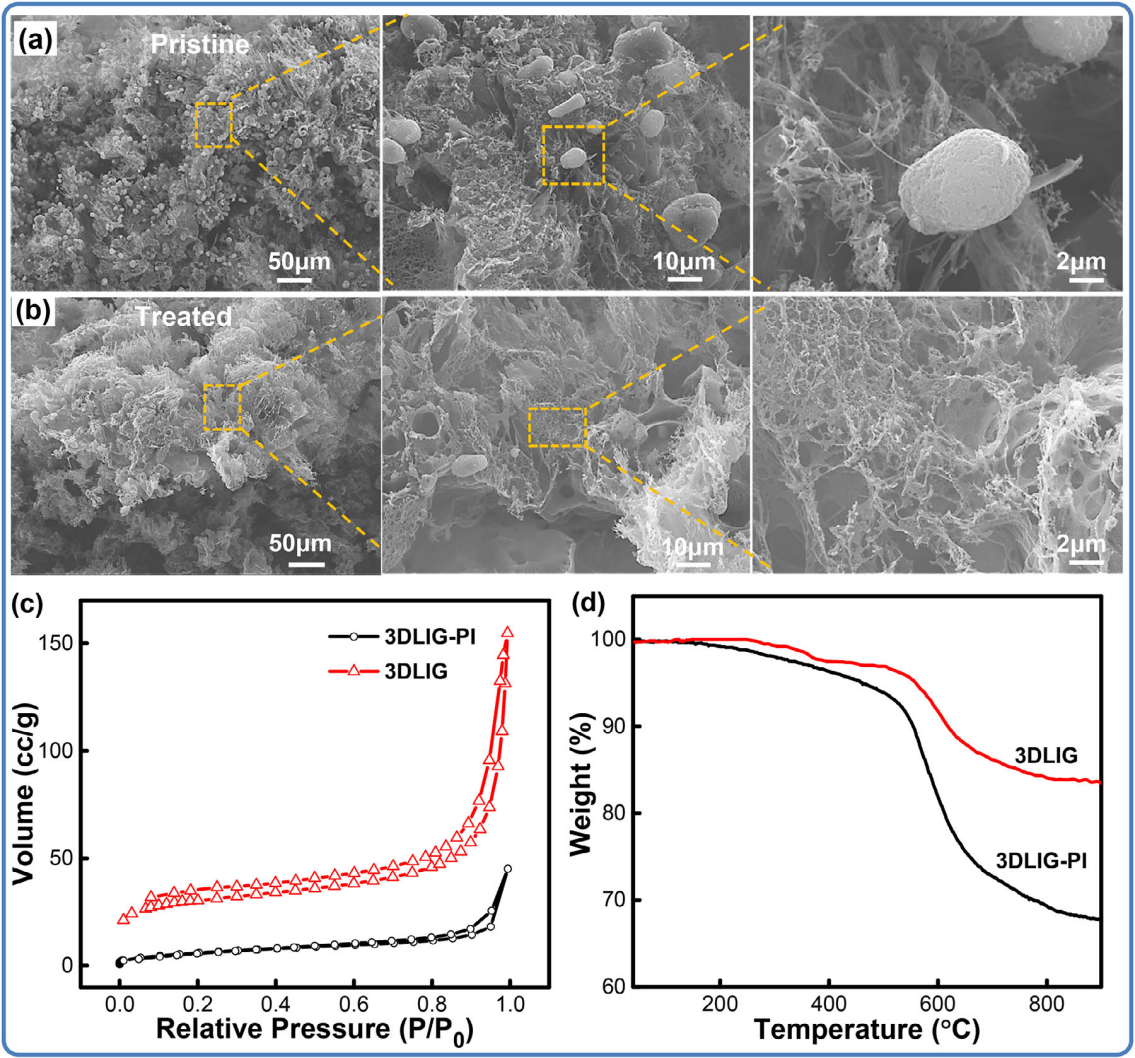
Morphological and spectroscopic characterizations of the pristine and treated 3D‐LIG foams. Cross‐section SEM images of the 3D‐LIG foams printed by the SLC technique (a) and the free‐standing specimen (b); N_2_ Adsorption‐desorption isotherms (c) and TGA curves (d) of 3D‐LIG‐based structures.

**Figure** **2**a depicts the cross‐section SEM images of the pristine 3D‐LIG foams and the treated ones. In comparison, residual sintered PI structures are clearly observed to intertwine with the LIG, while the surface morphology of treated specimens exhibits porous network in a micron‐scale in Figure 2b. From the adsorption‐desorption isotherms in Figure 2c, both 3DLIG‐PI and 3D‐LIG foams exhibit hysteresis loops, indicating a mesoporous material characteristic. That of the 3D‐LIG foam reflects a more complex pore structure and a wider pore size range, including micropores, as shown in Figure S1a,b (Supporting Information). BET analysis further confirms the formation of porous networks, with a tremendous increase in the specific surface area from 22 to 112 m^2^ g^−1^. The XPS characterizations have been carried out and comparable to analyze the contents and determine the surface elemental composition. From the XPS spectra in Figure S1c (Supporting Information), each sample presents a primary C 1s at 284.2 eV, O 1s at 532.0 eV, and N 1s at 400.4 eV, along with an increase in the intensity of C1s in the treated specimen. Furthermore, the high‐resolution spectrum C1s can be decomposed into four peaks, including the main peak of sp^2^ C─C at 284.0 eV, which is a characteristic peak of graphene,^[^ ^42^ ^]^ and the other three peaks of sp^3^ peak at 284.8 eV, C─O peak at 286.3 eV, and C = O peak at 288.4 eV, as shown in Figure S1d (Supporting Information). From the TGA analysis in Figure 2d, both curves display the major weight loss between 500 °C and 800 °C, which can be attributed to the decomposition and carbonization of residual PI particles. As shown, the free‐standing 3D‐LIG foam exhibits a high thermal stability with a relatively slow and gradual weight loss, retaining over 85% weight even at 800 °C. In comparison, the 3DLIG‐PI foam shows more pronounced weight loss (dropping to ≈75% at 400–600 °C), indicating a lower thermal stability in this range, possibly related to the decomposition of PI particles. The TGA data further confirm that PI structures have been successfully removed in the treated foam. Meanwhile, the treated free‐standing foam with a high thermal stability tends to have a better flame‐retardant behavior as it can resist decomposition and flammable gas release. In Figure S1e,f (Supporting Information), Raman spectra and XRD have also been applied to characterize the crystalline quality of free‐standing foams,^[^ ^43^, ^44^ ^]^ in which the presence of the typical 2D peak in Raman and the peak at 25.9° in XRD are highly consistent with the graphene.^[^ ^45^ ^]^

By virtue of the interconnected macroporous networks of the free‐standing foams, various polymer matrixes have been proven to be available for the development of 3D‐LIG composites, such as epoxy, rubbers, paraffin, thermoplastic polyurethane, and some macromolecules. The characteristics of LIG structures and polymers significantly play a dominant role in governing the structure‐property relationships of the resulting composites.

Here, the rigid and flexible matrices‐based composites were prepared to investigate the mechanical property, electrical properties, and potential applications. Taking the resin matrix as rigid fillers, Figure S2a (Supporting Information) displays the obtained wing model, the gear, and star samples with multiscale features. Figure S2b (Supporting Information) exhibits the designed composites by infiltrating Ecoflex into the 3D‐LIG foams under vacuum, including the kirigami‐structured, honeycomb‐structured, and cylinder specimens. Figure S3 (Supporting Information) highlights the SEM images of 3D‐LIG/epoxy composites with varied structures, showing the excellent resin adhesion of the graphene sheets. At the resin content of 10 wt%, the surface presents a glossy appearance and retains the porous interconnected networks to provide continuous pathways electrically (Figure S3a, Supporting Information). As the content increases, the pores of the LIG foams are gradually infiltrated, and the graphene flakes are randomly covered by the epoxy matrix, as shown in Figure S3b,c (Supporting Information). Similarly, 3D‐LIG/Ecoflex composites with different microstructures have been synthesized. SEM images in Figure S4 (Supporting Information) show fish‐scale‐like features, which reveal the interconnected networks of LIG within the nanocomposites. As the content of Ecoflex increases from 40wt% to 70wt%, the contour of LIG cells gradually fades and forms a smooth surface.

**Figure 3:**
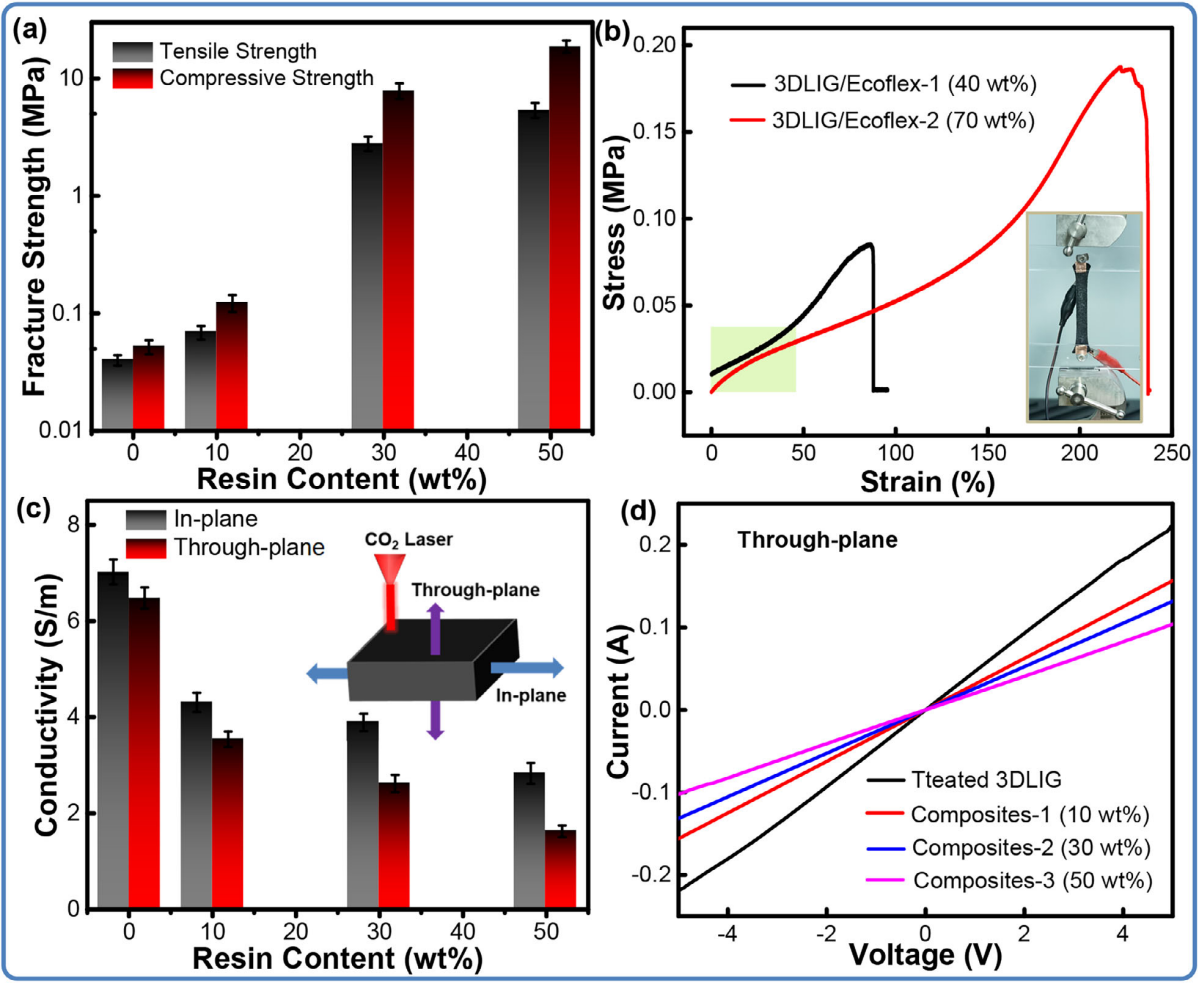
Mechanical and electrical performance of the 3D‐LIG/polymer composites. (a) Mechanical strength of the 3D‐LIG/epoxy composites; (b) Tensile fracture stress‐strain curves of the 3D‐LIG/Ecoflex composites; Electrical performance (c) and current–voltage curves (d) of the 3D‐LIG/epoxy composites.

The efficient synergy of LIG foams and polymers has enabled a boost in both mechanical and electrical properties of the 3D‐LIG composites. The addition of resin leads to an obvious increase in mechanical properties as systematically summarized in **Figure** **3**a. When the resin concentration varies from 0 to 50 wt%, the tensile strength increases from ≈71 kPa to ≈5.4 MPa by a 7606% improvement with a high specific strength of 6.8 × 10^3^ (N m)/kg^−1^, and the compressive strength increases linearly from ≈33 kPa to ≈18.8 MPa with a 56 969% enhancement. This indicates that the composite has an interconnected 3D structure infiltrated with epoxy, where the epoxy serves as the reinforcement. As described in Figure S3 (Supporting Information), with the increase of the epoxy content, the porous networks of the 3D‐LIG foam are gradually filled by the polymer matrix, showing a smooth appearance similar to that of pure epoxy. During the loading process, the networks of the 3D foam allow for load transfer between the polymer matrix, which results in a largely improved mechanical strength compared to the pristine foam. The stress‐strain curves in Figure S5 (Supporting Information) reveal the failure process of the composites upon loading, reflecting a typically brittle fracture behavior. Given the excellent electrical and mechanical performance, the functionalized composites exhibit both the dielectric property and Joule‐heating property, and can be expected for structural parts, wave‐absorbing materials, and thermal interface materials.^[^ ^46^ ^]^

The role of 3D‐LIG foams in flexible and stretchable composites has also been developed and systematically discussed in rubber‐enhanced composites. Figure 3b points out the tensile behavior of the composites, and the stress‐strain curves present the typical elastic, plastic, and failure stages. The effort of the flexible rubber has stepped up the mechanical behavior of 3D‐LIG foams. With the increasing addition of Ecoflex from 40 to 70 wt%, the ultimate tensile stress of the composites increases from ≈0.08 to ≈0.18 MPa and the strain improves from 86% to 230%. At the content of 40 wt%, the composites show a 50% higher linear elastic region corresponding to an elastic modulus of ≈0.1 MPa. Figure S6 (Supporting Information) tests the mechanical property of the flexible composites and gives the stress‐strain curves under 10–80% compressive strain (Figure S6a, Supporting Information), exhibiting a large strain deformation (≥90%) and stable recoverability. As shown in Figure S6b (Supporting Information), the continuous compress‐release deformation process at a 80% strain with 10 cycles reflects the negligible degradation of stress and a good mechanical robustness. Meanwhile, integration of LIG foams as a conductive scaffold endows the composites of through‐plane conductivity varying from 3.54 to 1.63 S m^−1^ and the in‐plane conductivity varying from 4.31 to 2.83 S m^−1^ with the resin content increasing from 10 to 50 wt%, as plotted in Figure 3c. Compared with the free‐standing 3D‐LIG foams, the through‐plane electrical conductivity of the composites decreases from 6.48 to 0.14 S m^−1^ due to the inertness of the inert Ecoflex. The I–V characteristic curves of the composites are systematically examined and suggest a constant resistance‐current behavior, as illustrated in Figure 3d. Notably, all of the curves show a remarkable linearity within the voltage range from −5 to 5 V. The improved electrical conductivity of the composites is attributed to the 3D interconnected LIG networks which provide more efficient paths for electron transfer inside the polymer and can be highly controlled by tuning the LIG content in the matrix.

### 2.3 Electric‐driven Performance of 3D‐LIG/Epoxy Composites for De‐icing and Wave‐absorbing

**Figure 4:**
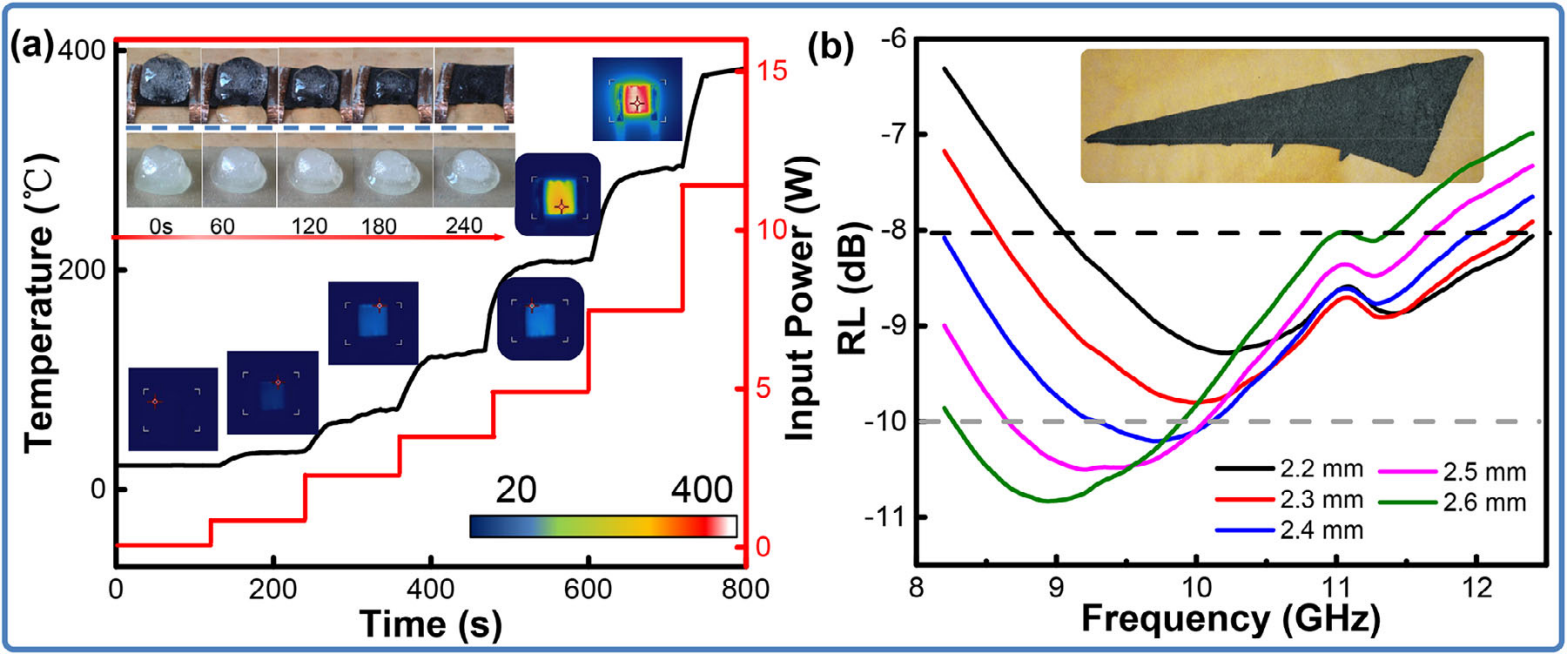
Functional performances of the 3D‐LIG/epoxy composites. (a) Joule‐heating performance and the de‐icing tests; (b) The wave‐absorption performance.

Based on the electrical conductivity and Joule‐heating performance of LIG,^[^ ^33^ ^]^ the 3D‐LIG/epoxy composites have been qualified as smart skins for thermal applications. **Figure** **4**a records the real‐time surface temperature variations of the composites as a function of input powers by the thermocouple. As shown, the temperature instantaneously increases with the electrical power applied, and when the electrical power remains constant, the temperature value gradually becomes stable. Specifically, an input power of 0.12 W successfully induces the composites from room temperature to ≈37 °C. When the power increases to 2.3 W, the surface temperature can quickly reach up to ≈145 °C. The infrared thermal images of Figure 4a also display the surface temperature variation with almost uniform heating distributions driven by different input powers, suggesting the formation of continuous electricity and heat‐conducting pathways in the composites. To demonstrate the electrothermal effect, the composites are utilized for efficient de‐icing. Specifically, an ≈8 cm^3^ sized ice cube is placed on the sample surface and a power of 4.8 W with the temperature of ≈230 °C is applied to melt it. As shown in the upper part of Figure 4a, the ice is completely melted after ≈6.5 min, and the composites still maintain intact without any damage, exhibiting the stable electric heating performance as thermal devices.

On account of excellent dielectric properties, the 3D graphene can also drive composites for microwave absorbing. The relationship between the reflection loss and frequency with different thicknesses of the 3D‐LIG/epoxy composite has been discussed. As illustrated in Figure 4b, we print a wing model with homogeneous structures by the fixed laser parameters and utilize a vector network analyzer to analyze the reflection loss (RL) across the 8.0–12.0 GHz range. The designed composites achieve a reflection loss below −8 dB over 3.1 GHz in the range of 8.9–12.0 GHz, suggesting an absorptivity of higher than 85%.^[^ ^47^ ^]^ The minimum reflection loss is registered of −10.8 dB at 9.1 GHz, with the effective absorption bandwidth (EAB) of 1.6 GHz within the 8.2–9.8 GHz range. It can also be found that the absorption intensity has been considerably enhanced with the increase of thickness. A possible reason for this result is that the formation of more interconnected networks can conduce to improve the propagated and reflected paths of microwave, enhancing the impedance and attenuation ability. Given the microwave absorption performance, the 3DLIG‐based composites have been validated as potential materials for radar stealth.

### 2.4 Piezoresistivity of 3D‐LIG/Ecoflex Composites for Motion‐monitoring

**Figure 5:**
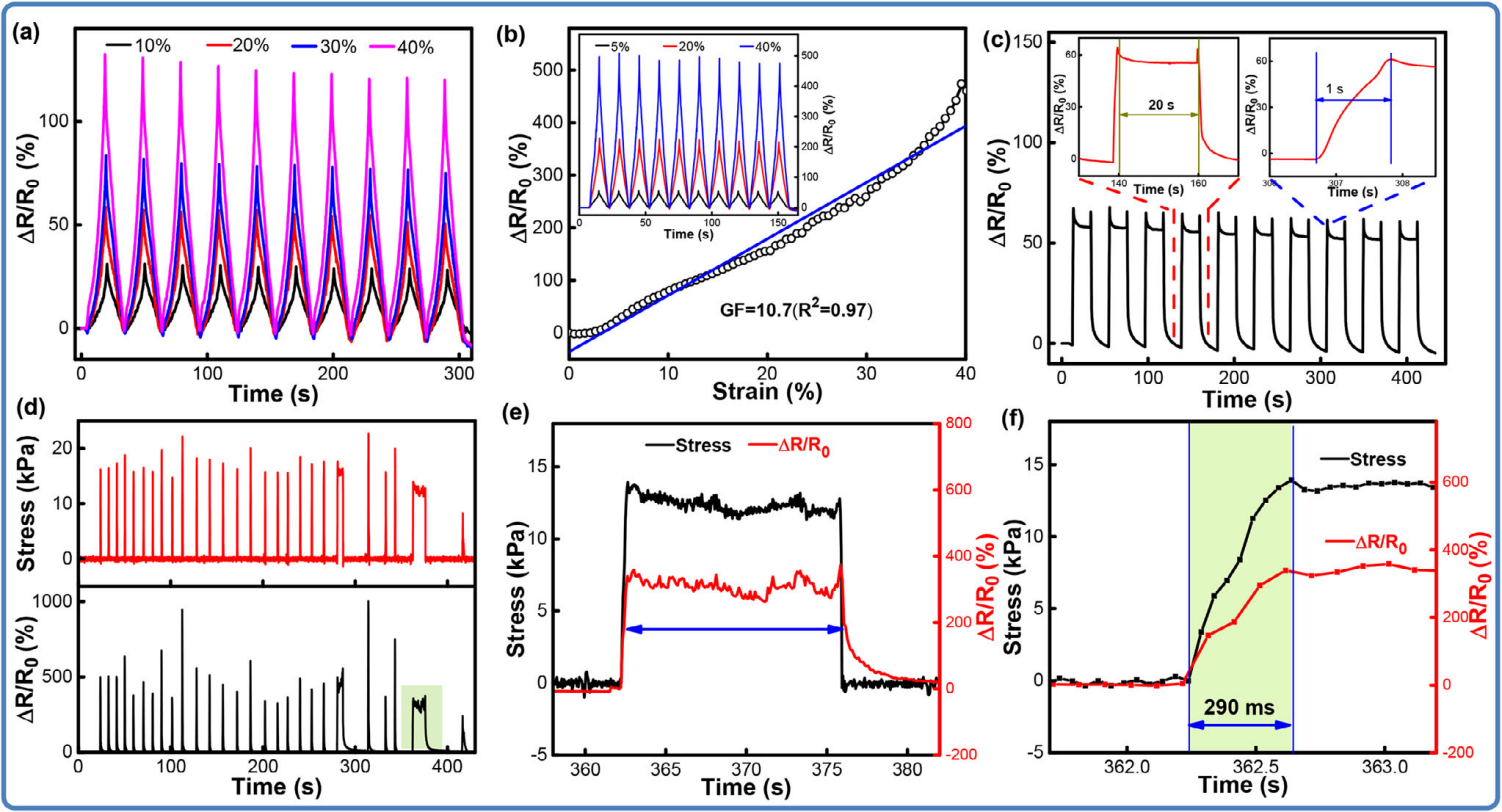
Piezoresistive characterizations of 3D‐LIG/Ecoflex composites. (a) Cyclic relative changes in resistance versus the applied strain from 10% to 40%; (b) The measurement of the sensitivity of the composites; (c) The resistance response at a programmed procedure; (d–f) Dynamic response and relaxation ability, and the corresponding sensing times.

Taking advantage of the excellent elasticity and conductivity, the 3D‐LIG/Ecoflex composites have been employed to evaluate the piezoresistive performance and the variation in electrical resistance is real‐time recorded using a digital multimeter equipped with a load cell under cyclic loading. **Figure** **5**a plots the recorded resistance changes of the flexible composites made by a 70 wt% content of Ecoflex under tensile strains. Obviously, the relative resistance (∆R) of the composites synchronously varies with these varying levels of strain, and gradually returns to the original value upon unloading. When subjected to the identical strains, the ∆R/R_0_ changes with the tensile strain in each cycle, and the maximum value remains highly consistent under dozens of cycles, thereby showing a remarkable stability and repeatability. Significantly, when the applied strain increases from 10% to 40%, the maximum ∆R/R_0_ synchronously increases from 30% to 120% with a uniform growth of 30% and a gauge factor (GF) value of ≈3.0, showing a linear and stable piezoresistive response. As the content of Ecoflex decreases, the resistance becomes more sensitive to the strain due to the fact that the conductive networks have been well interconnected. As depicted in Figure 5b, the sensitivity and linearity of the piezoresistive response have been investigated. The recorded data plot a nearly linear trend with the strain increasing from 0% to 40%, and a GF value of ≈10.7 has been obtained by calculating the slope of the fitted line. The inset picture displays the relative change in resistance (ΔR/R_0_) under different tensile strain loads of 5%, 20%, and 40%, respectively. In each cycle, the responsive variation of the relative resistance synchronizes with the applied strain, and no obvious value distortion or drift takes place, suggesting a highly linear and consistent piezoresistivity. The mechanism behind the sensing of the strain sensor may be attributed to the slippage of the recoverable conductive networks (marked in Figure S4, Supporting Information) that guarantee the excellent sensing performance during tension‐release cycles. The pore structures enable the composites to have a wide working range of 0–40% and the well repeatability.^[^ ^48^ ^]^The step response of the sensor is analyzed by applying a programmable strain of 5% with a fixed loading time (1 s) and holding time (20 s). As plotted in Figure 5c, the change in resistance maintains a well‐stimulated regime and ultimately returns to the initial value when the stress is released, reaffirming the reliability and consistency of the flexible composites. To test the dynamic and instantaneous sensing ability, Figure 5d further gives the real‐time response of the sensor when subjected to the transient and abrupt stress. The results reflect that the sensor responds quickly under different stress and the ΔR/R_0_ reaches a maximum value of 998% under a 22.7 kPa loading. Figure 5e shows that the output resistance signal of the sensor is well fitted with the varied stress, suggesting that the input of stress is highly tracked via the output value of resistance. The ability to detect subtle movements or vibrations is reflected in the fast response time of 290 ms at an instantaneous 13.9 kPa tension‐stress for the flexible strain sensor in Figure 5f. The mechanism behind the sensing behavior is the variation in contact area due to the slippage of graphene sheets, thereby inducing in enhanced sensing signals. And the recoverable conductive networks (marked in Figure S4b, Supporting Information) in the composites guarantee the excellent sensing performance during tension‐release cycles.

**Figure 6:**
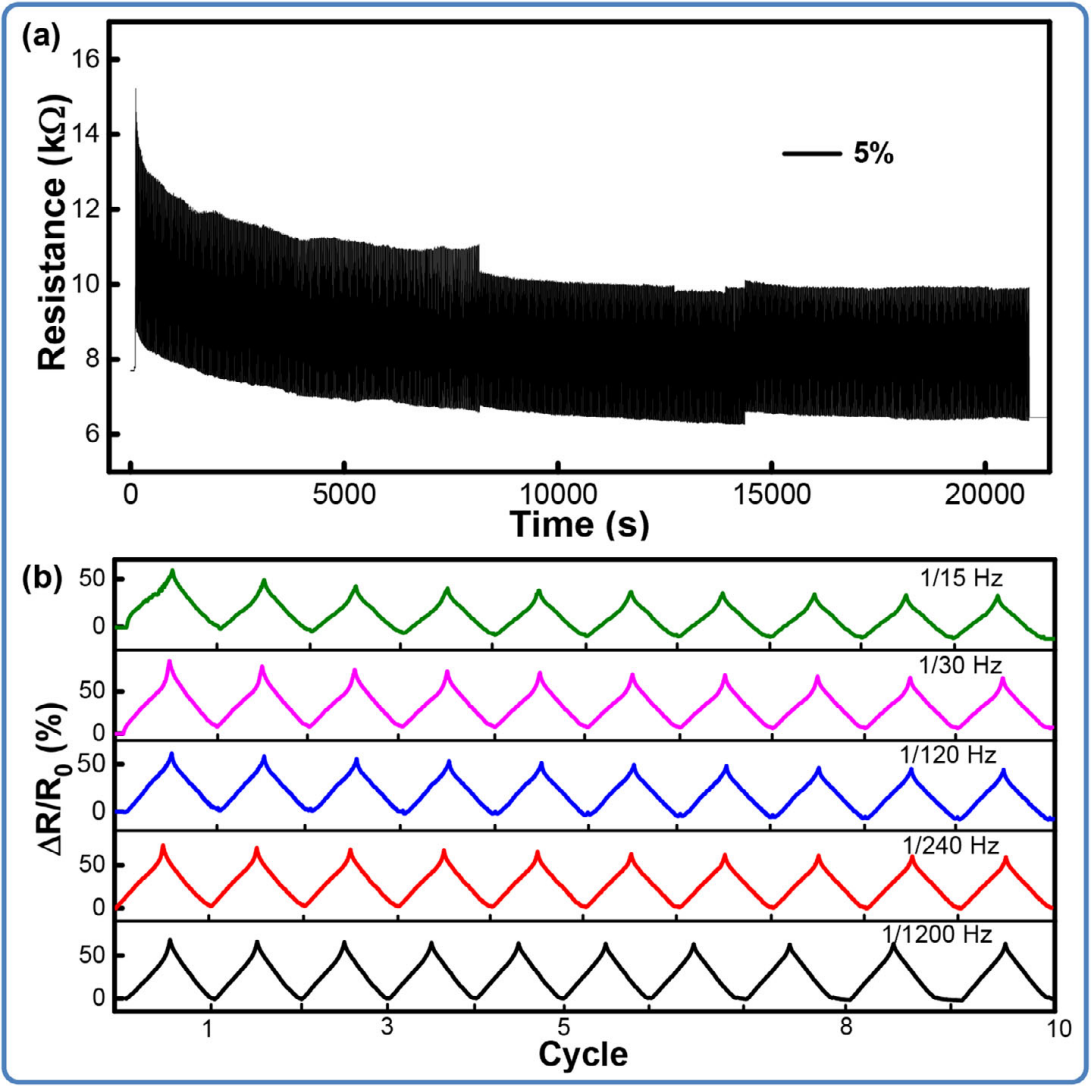
Dynamic stability and robustness of the flexible sensor. (a) The variation in resistance by applying and releasing tension for 1500 cycles; (b) The piezoresistive response under cyclic tensile strain at various frequencies.

To further assess the reliability and feasibility upon identical strain loading, the cycling stability has been evaluated by applying 1500 repeated stretching/releasing cycles at a strain of 5% in **Figure** **6**a. From the illustration, the obtained resistance signals present a progressive decline, while the relative variation almost remains constant, reflecting an excellent piezoresistive sensitivity. It can also be observed that the resistance gradually stabilizes without severe degradation, showing a good durability of sensing ability. Figure 6b also verifies the stability that the ΔR during the releasing stage aligns appropriately with that recorded during the stretching stage, and the value is nearly independent with the frequencies changing from 1/15 to 1/1200 Hz under stretching/releasing cycles from 0% to 5% strain.

**Figure 7:**
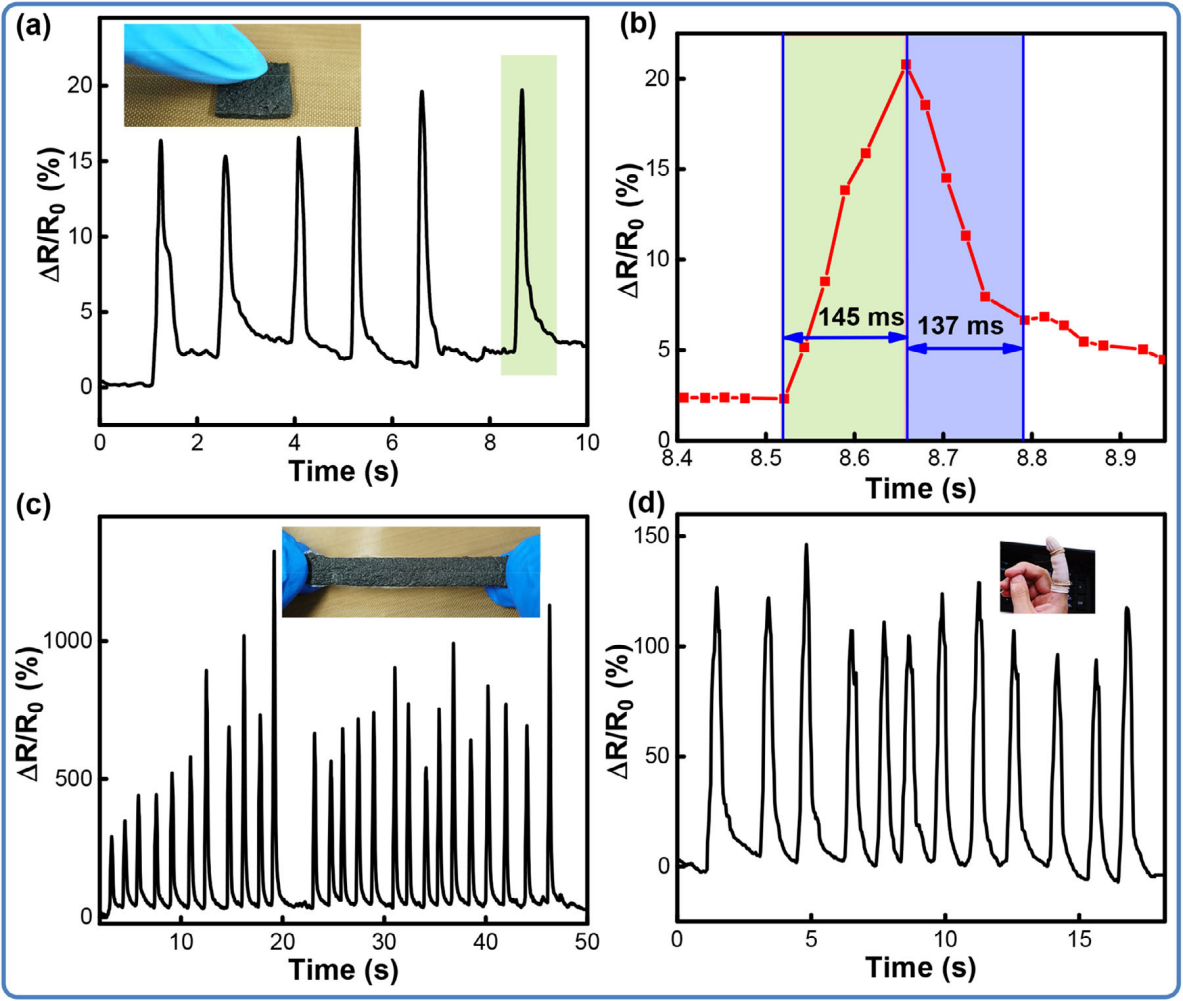
Application of the fabricated flexible strain sensor for real‐time monitoring of human subtle motions. (a) Finger pressure and release; (b) Response time and recovery time; (c) Instantaneous stretching response; (d) Finger flexion and extension.

Both the credible stability and wide linear workspace bring great convenience for the application of the flexible sensor, enabling human motion capturing and analyzing. **Figure** **7** exhibits the applicability of the flexible composites prepared as an e‐skin in motion detecting of pressing, bending, and stretching deformations. Figure 7a demonstrates the response of the e‐skin to the finger pressure, showing a stable response. Figure 7b further displays the dynamic response and relaxation time of the sensor. With the fast response ability, the time intervals of the pressing and releasing stages are recorded as 145 and 137 ms, respectively. The response to the instantaneous stretching is presented with repeatable large‐scale sensing performance (Figure 7c). Nevertheless, the finger bending has also been real‐time monitored, as shown in Figure 7d. The flexible sensor is attached to the forefinger joint to monitor the finger‐bending motions. Obviously, the relative resistance immediately increases during finger flexion while recovering to the initial value upon extension. And subsequent to several frequent cycles, the sensor still faithfully registers the real‐time resistance change for finger bending and extension. These interesting findings displayed by the 3D‐LIG/Ecoflex sensor highlight the remarkable capability for motion monitoring, thereby reinforcing its standing in flexible wearable devices.

## 3 Conclusion

In summary, an effective strategy for preparing graphene‐based functional composites was introduced, where the polymer matrix were infiltrated into the free‐standing 3D‐LIG foams which were prepared by a 3D printing technique, achieving significant enhancement in mechanical and electrical performance. Introducing the epoxy led to 3D‐LIG/epoxy composites with a through‐plane electrical conductivity of ≈3.54 S m^−1^ and a compressive strength of ≈18.8 MPa, a remarkable ≈56 969% improvement over pristine 3D‐LIG foams. Meanwhile, the flexible rubber endowed the as‐synthesized composites with a 230% tensile‐failure strain and a 50% high linear elastic strain. Leveraging these improved mechanical and electrical properties, 3D‐LIG/epoxy composites exhibited the practical versatility for de‐icing and absorption of radar waves, with the absorptivity > 85% in 8.9–12.0 GHz. Similarly, the flexible 3D‐LIG/Ecoflex composites, with the reversible conductive networks, have functioned as a piezoresistive sensor, showing a 0–40% wide linear strain range and a 137 ms fast response time. The proposed 3D graphene‐based composites fabrication method was simple, scalable, and efficient enough to make a contribution to the development and application of conductive composites. For future work, we will focus on optimizing the interfacial interactions between the LIG foams and polymer matrices to further tailor the properties, thus exploring the feasibility and durability of these composites for potential applications in diverse environmental conditions.

## 4 Experimental Section

### Preparation of 3D‐LIG Foams

The thermoplastic PI powders with a size of ≈ 10 µm were prepared as the raw carbon sources, and then were irradiated using a CO_2_ laser with a controlled XYZ‐movement platform (DLS 2.3, Universal Laser Systems, Inc.). Typical value of laser power was fixed at 1 W with the pulses per inch set at 500 and the scanning speed at 50 mm s^−1^. The powder‐feeding thickness was selected at 50 µm. The powder spreader (BEVS 1806, BEVS Industrial Co., Ltd.) with a resolution of 10 µm was used to adjust the layer thickness. DMF solvent (Macklin Reagent Co., Ltd) was employed to remove the residual powders at 120 °C for 3 h. If necessary, a heat treatment at 150 °C was adopted to further dissociate the residual solvent.

### Preparation of 3D‐LIG/Polymer Composites

Ecoflex0030 (Smooth‐On, Inc.) and Epoxy (AG80) were utilized to functionalize the 3D‐LIG foams. Isopropyl alcohol and acetone were employed to dilute the Ecoflex and epoxy, respectively. Using the vacuum‐assisted impregnation method, the 3D‐LIG foams were immersed in the prepared polymers for 1 h at one time. And then the composites were obtained by heating at 80 °C for 3 h to cure the polymer and remove the solvent.

### Structural Characterization

Morphologies were characterized using SEM with a JEOL JSM 7001F at 10 kV to examine. Raman spectra were collected with a Horiba HR800 Raman microscope using a 532 nm laser with a power of 5 mW. XRD characterization was performed on Rigaku D/max 2550. XPS measurements were performed with an ESCALAB 250Xi instrument. TGA analysis (STA 449F3, NETZSCH Company) was carried out at 10 °C min^−1^ under nitrogen. Specific surface area was tested by a McMuratik ASAP2460 BET surface analyzer.

### Performance Evaluation

Mechanical strength was measured by a dynamic mechanical analyzer (DMA) (Q850, TA Instrument Inc.). The conductivity and piezoresistivity were quantified using a Keithley 2450 Sourcemeter. Microwave absorption properties were measured by Vector Network Analyzer (VNA, E5071C).

## Conflict of Interest

The authors declare no conflict of interest.

## Supporting information



Supporting Information

## Data Availability

Data sharing is not applicable to this article as no new data were created or analyzed in this study.
