# Supplementary material for: Laser‐Induced 3D Graphene Enabled Polymer Composites with Improved Mechanical and Electrical Properties Toward Multifunctional Performance
Source: Adv Sci (Weinh). 2025 Sep 3;12(43):e09039. doi: 10.1002/advs.202509039 (PMC12631839; doi:10.1002/advs.202509039)
Supplement: Supplementary file 1 — Supporting Information [file ADVS-12-e09039-s001.docx]

Supporting Information

**Laser-induced 3D graphene enabled polymer composites with improved mechanical and** **electrical properties toward multifunctional performance**

*Fu Liu, Sida Luo, Jingyang Li*, Zhe Wang, Xu Wang, Wenqian Hao, Yanan Wang*


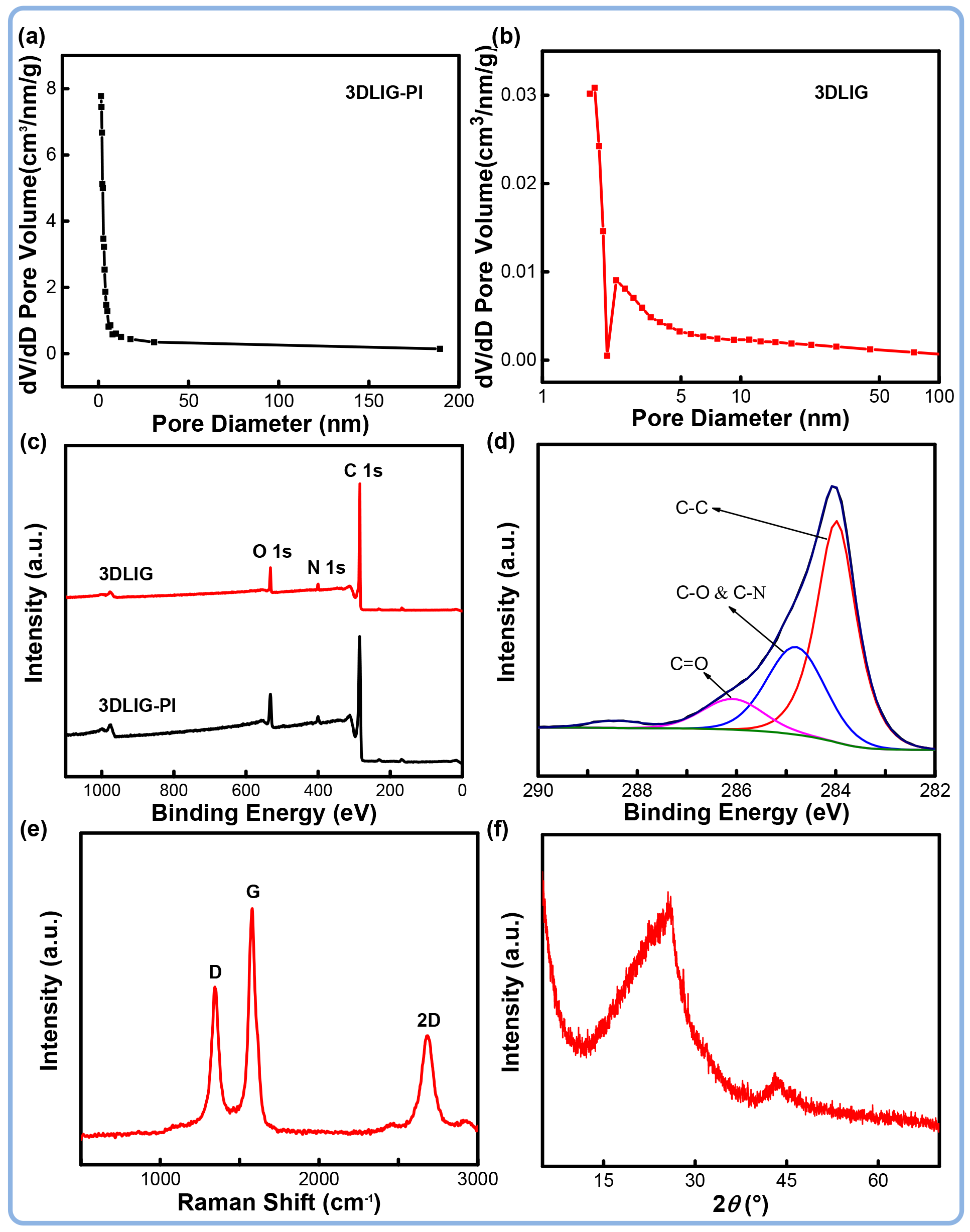


**Figure S1.** Structural properties of the as-prepared 3D-LIG foams. The pore size distribution curves of the 3DLIG-PI foam (a) and free-standing foam (b); (c) XPS surveys of the 3DLIG-PI foam and free-standing foam; (d) C 1s of the free-standing 3DLIG foam; (e) Raman spectra and (f) XRD pattern of the free-standing foams.


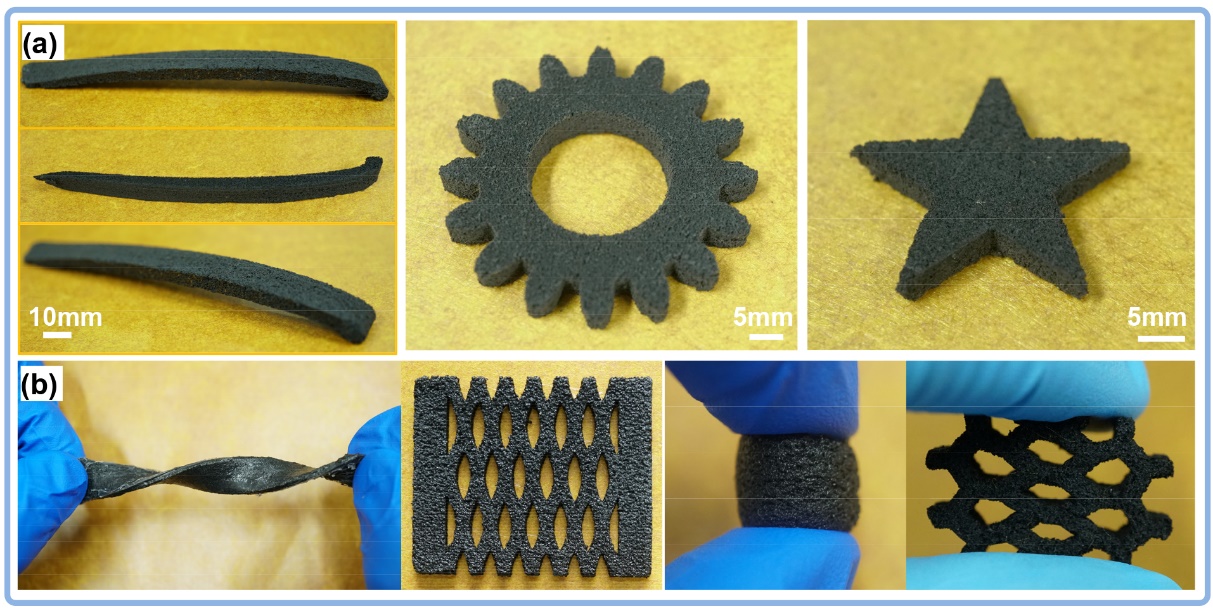


**Figure S2.** Photographs of the 3DLIG/polymer composites. (a) A wing model, a gear model, and a five-pointed star model built by the 3DLIG/epoxy composites; (b) Kinds of designed structures with desired features constituted of 3DLIG/Ecoflex composites.


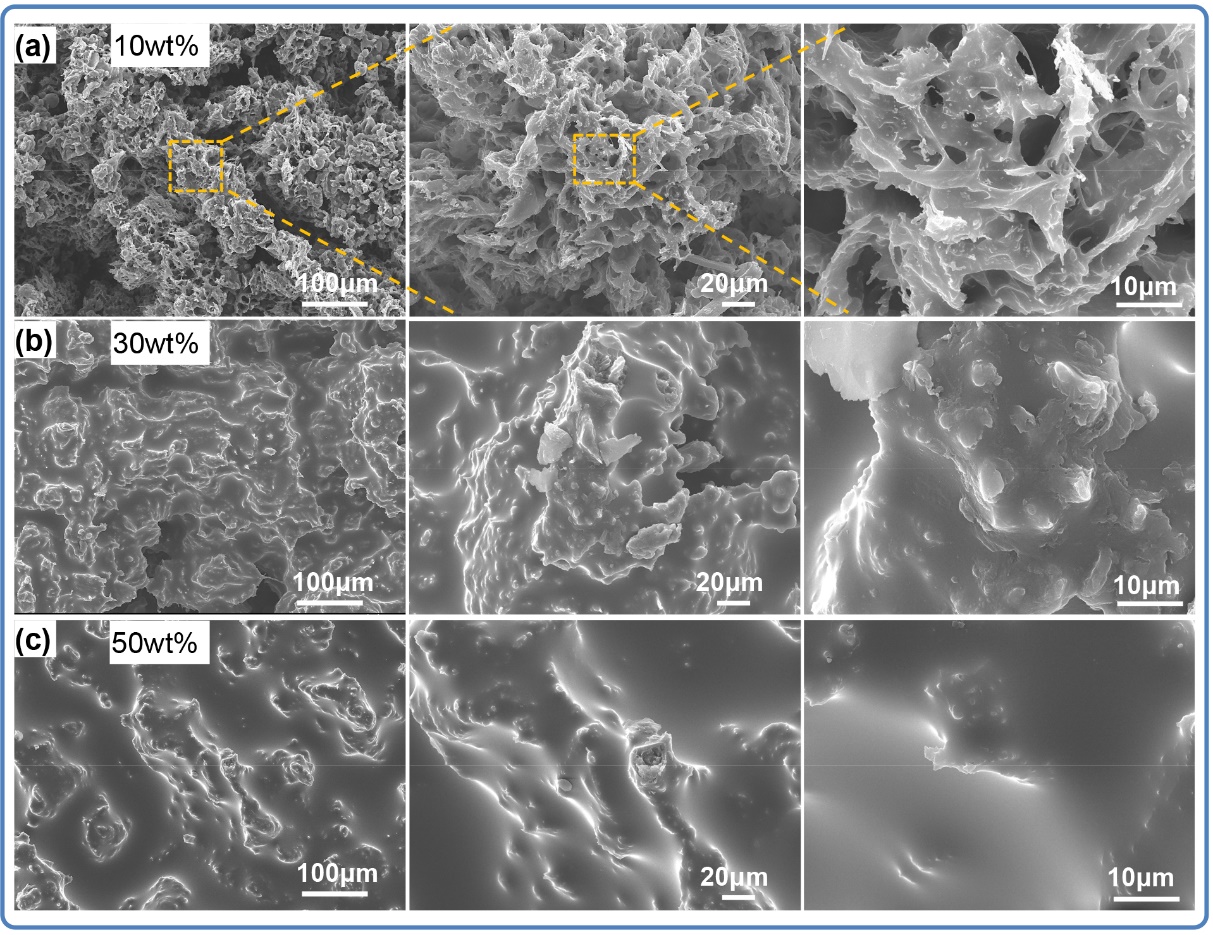


**Figure S3.** SEM images of 3D-LIG/epoxy composites produced by different resin concentration of 10wt% (a), 30wt% (b) and 50wt% (c).


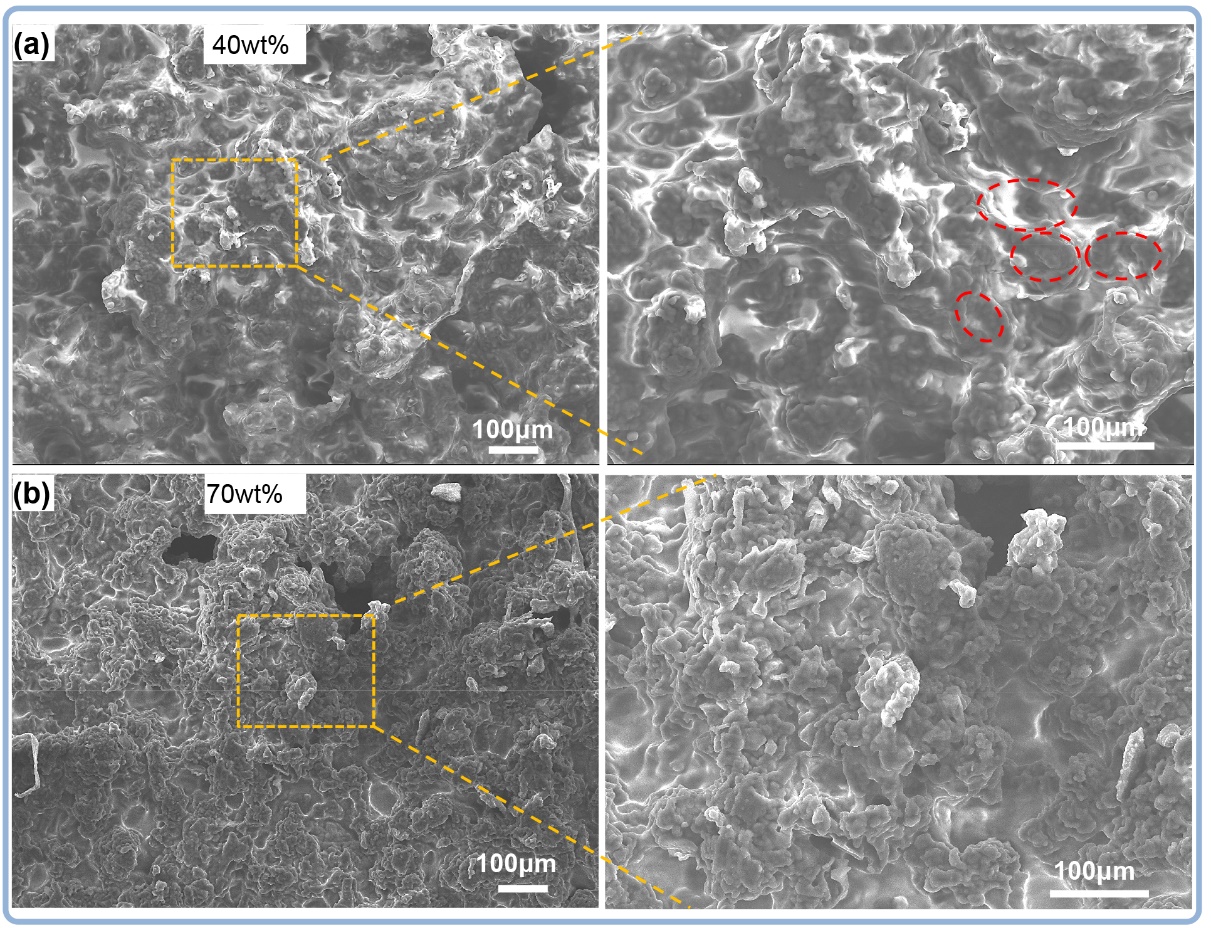


**Figure S4.** SEM images of 3D-LIG/Ecoflex composites produced by different resin concentration of 40wt% (a) and 70wt% (b).


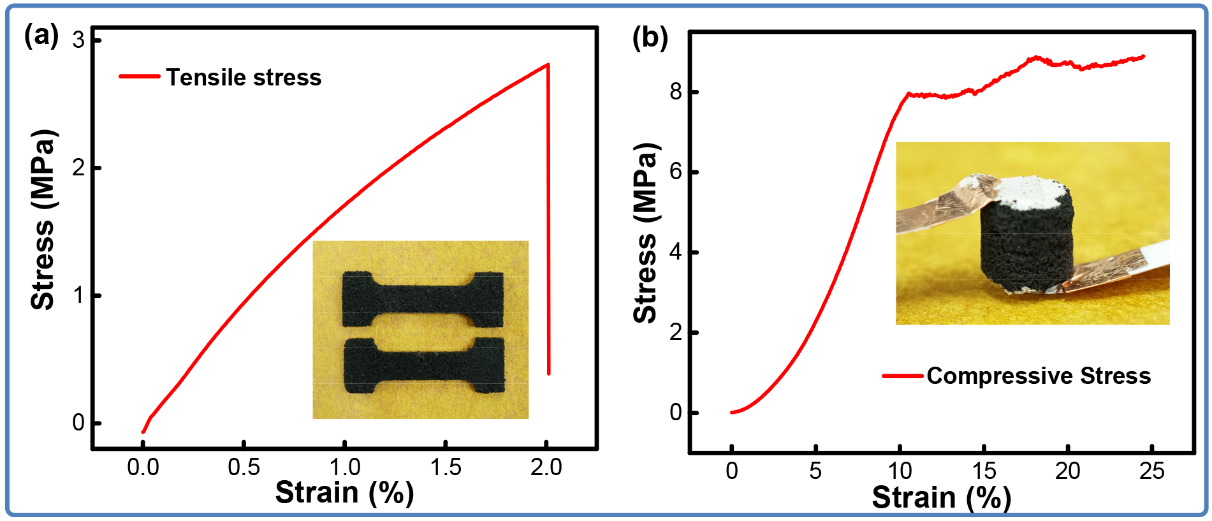


**Figure S5.** Mechanical characterization of the 3D-LIG/epoxy composites. The tensile fracture curve (a) and the compressive fracture curve (b).


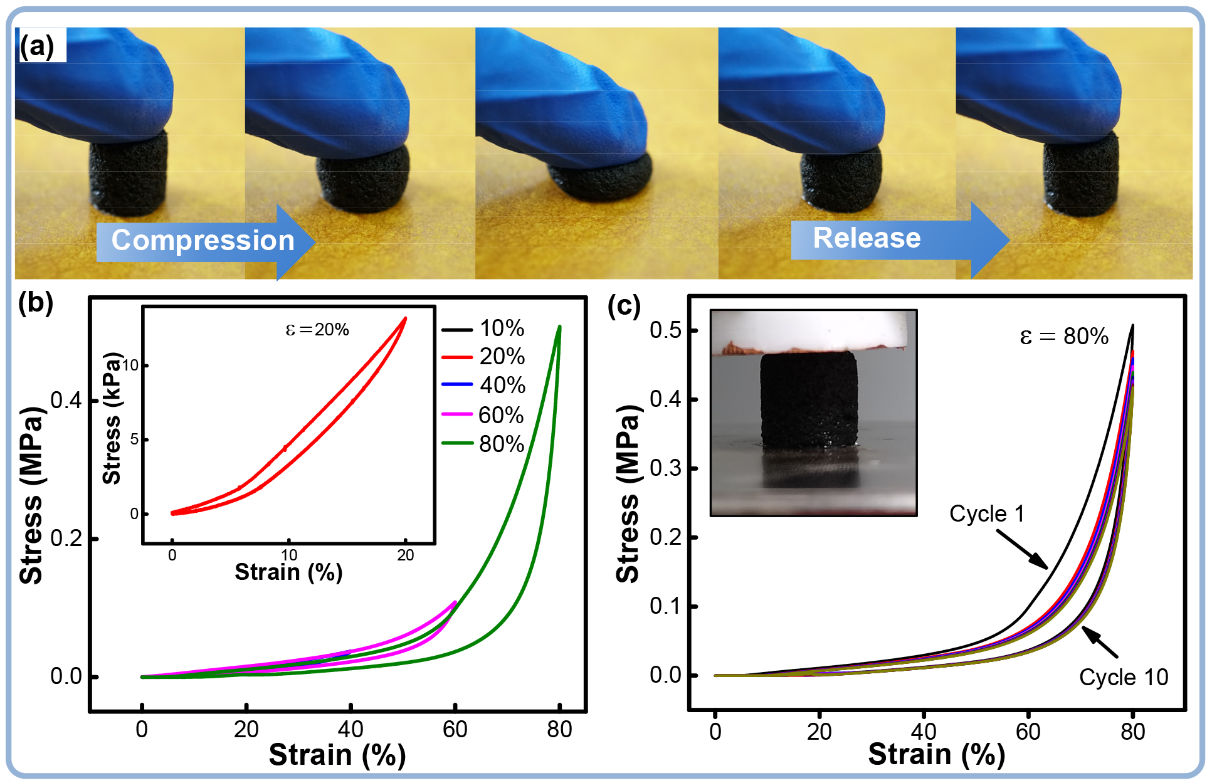


**Figure S6.** Mechanical characterization of the 3D-LIG/Ecoflex composites. (a) The compression-release process of the 3DLIG/Ecoflex composites; (b) The stress-strain curves with strains from 10% to 80%; (c) The cyclic stress-strain curves under a strain of 80%.
